# Working and Caring During the Coronavirus Pandemic: The Impact of Workplace Policy on Working Female Caregivers

**DOI:** 10.1093/geroni/igab046.3546

**Published:** 2021-12-17

**Authors:** Jessica McLaughlin, Ashley Taeckens-Seabaugh, Amy Kennicutt, Taylor Capellaro

**Affiliations:** 1 University of Denver, Fort Collins, Colorado, United States; 2 University of Denver, Denver, Colorado, United States; 3 Data 4 Equity, LLC, Miami Beach, Florida, United States; 4 Connally Counseling, Garden City, Michigan, United States

## Abstract

The unprecedented nature of the coronavirus pandemic created significant socioenvironmental changes for working caregivers who found themselves juggling a new landscape of working and caring. Changes in workplace policy were often intended to accommodate those with caring responsibilities, however, there is little information available on how working female informal caregivers of older adults (defined as individuals age 50 or older) received, interpreted, and experienced those policy changes. Given this, it is necessary to gather a complete picture of workplace policy in the daily lives of working female caregivers during the pandemic. This qualitative study involved interviews held between February and April 2021 via video conferencing technology with 29 working female caregivers, ranging in age from 27 to 75 years old. Using a Role Conflict framework and descriptive, structural, and emotion coding strategies, analysis of written transcripts revealed that, while many caregivers were grateful that their workplaces had become more accommodative during the pandemic, apprehension and uncertainty about the future, both with caregiving and with work, also weighed heavily on many of them. The most positively endorsed workplace policy changes were flexibility in work schedules and the ability to work remotely during the pandemic. This research elucidates policy implications for working female caregivers outside of the pandemic context, as many of these policies enabled caregivers to provide care while working with greater ease.

